# Validation of the Italian version of the abbreviated expanded prostate Cancer index composite (EPIC-26) in men with prostate Cancer

**DOI:** 10.1186/s12955-019-1214-x

**Published:** 2019-08-29

**Authors:** Chiara Marzorati, Dario Monzani, Ketti Mazzocco, Marianna Masiero, Francesca Pavan, Massimo Monturano, Gabriella Pravettoni

**Affiliations:** 1 Department of Oncology and Hemato-Oncology, University of Milan, Milan, Italy; 2 Applied Research Division for Cognitive and Psychological Science, European Institute of Oncology IRCCS, Milan, Italy; 3 Department of Biomedical and Clinical Sciences, University of Milan, Milan, Italy; 4 Patient Safety & Risk Management Service, European Institute of Oncology IRCCS, Milan, Italy

**Keywords:** Prostate cancer, Epic-26, Quality of life, Validation, Decision-making

## Abstract

**Background:**

This study aims to validate and evaluate the psychometric properties and reliability of the Italian version of the Expanded Prostate Cancer Index Composite – Short Form (EPIC-26), a measure of quality of life (QoL) for prostate cancer patients.

**Methods:**

Two hundred and eighty-four prostate cancer patients completed the Italian version of the EPIC-26 questionnaire at 45 days (T1) and 3 months (T2) after robot-assisted radical prostatectomy (RARP). Psychometric properties were evaluated using structural equation modeling: the goodness of fit of the correlated five-factor model (CFFM) for the EPIC-26 was assessed using the confirmatory factor analysis (CFA), while longitudinal invariance was conducted to assess the ability of the EPIC-26 to measure QoL construct over time. Test-retest reliability was assessed as well by considering intraclass correlations.

**Results:**

At T1, the CFFM model displayed a good fit to data. Similarly, the model showed an adequate fit also at T2. Results of the reliability analysis attested the acceptable internal consistency and test-retest reliability of each dimension: all Cronbach’s alphas could be classified as acceptable (i.e., above .65) except for low Cronbach’s alpha for hormonal dysfunction at T1 (i.e., .638) and urinary irritation at both waves. (i.e., respectively .585 and .518). Finally, psychometric properties were invariant over time and each of the five dimensions of QoL displayed from moderate (all ICCs above .500) to good test-retest reliability (i.e. ICC for urinary incontinence = .764).

**Conclusions:**

Results of the CFA and the measurement invariance analysis demonstrated the validity of the Italian version of the EPIC-26 to assess QoL in prostate cancer patients. Its reliability and good psychometric qualities are well-supported, thus providing a valid tool to assess health-related quality of life and its change over time in prostate cancer patients.

## Background

Prostate cancer is one of the most common cancers in men with almost 70% of the cases occurring in the developed countries, where advances in screening and treatments have led to an increase in early tumor detection and a prolonged patient lifespan [1, 2]. Despite these advances, prostate cancer patients report a worsening of their quality of life (QoL) [3–5]. Indeed, radiotherapy and invasive surgery can cause urinary incontinence, sexual problems and bowel dysfunction, and they are often related to distress, anxiety or fatigue [6–9]. In this vein, patient-reported outcomes (PROs) play an important role in the process of care of prostate cancer patients who have to deal with both functional and psychological problems [5, 7, 8]. A recent systematic review showed that, among the great availability of prostate-cancer specific questionnaires measuring PROs, the Expanded Prostate Cancer Index Composite (EPIC) is the most suitable cancer-specific survey in urology departments to measure patient’s physical and psychological well-being [10]. Through the “Evaluating Measures of Patient-Reported Outcomes” (EMPRO) tool, the EPIC obtained, along with the University of California Los Angeles-Prostate Cancer Index (UCLA-PCI), the highest score in terms of concepts and population intended to assess, and very high scores in validity, interpretability, and responsiveness. Moreover, EPIC was also recommended because it is the only questionnaire investigating hormonal and irritative/incontinence urinary dysfunction domains. The original version of EPIC is composed of 50 items and is developed by Wei and colleagues [11]. Considering the difficulty of administering the questionnaire during clinical practice, a short-version was introduced composed of 26 items. The new version, named EPIC-26, is the most used brief self-report scale and it has already been validated in Norway, USA, China and Germany [12–15].

Its administration allows physical and psychological information to be collected on specific dimensions, as urinary incontinence, urinary irritation, bowel, sexual and hormonal dysfunction, scored from 0 (worst) to 4 or 5 (best). All domains of EPIC-26 are highly correlated with all domains of the longer version EPIC-50 (r ≥ 0.96) [12–15].

**Fig. 1:**
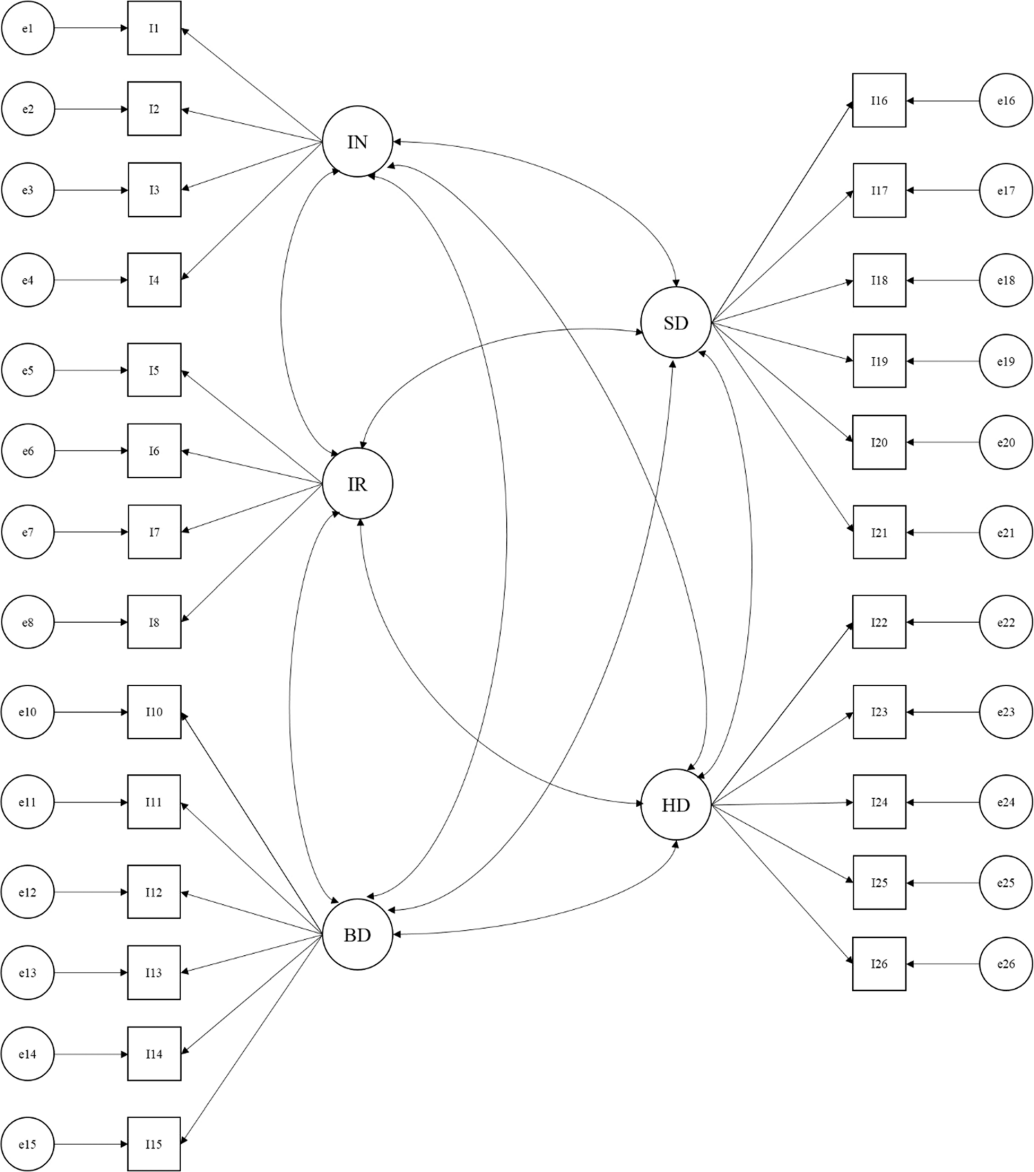
The correlated five-factor model. Note: IN = Urinary incontinence; IR = Urinary irritation; BD = Bowel dysfunction; SD = Sexual dysfunction; HD = Hormonal dysfunction

The proposed factor structure for the EPIC-26 is a correlated five-factor model (CFFM) [15, 16]. As shown in Fig. 1, urinary incontinence and urinary irritation are both measured by four items; bowel and sexual dysfunctions are both measured by six items, while five items measure hormonal dysfunction. A single item (i.e., item 9) measuring overall urinary symptomatology is a stand-alone item and is not included in any of the domains because it overlaps on both urinary incontinence and urinary irritation.

High internal consistency and test-retest reliability - Cronbach’s alpha ≥0.70 and r ≥ 0.69 respectively - have been reported in all the domains [15–17]. These psychometric properties, along with being less time-consuming and easier to use than the full version, encouraged the use of the EPIC-26 in clinical and research setting over time.

Considering the pivotal role of the EPIC-26 to assess QoL in prostate cancer patients [10], the main aim of this study was to develop an Italian version of this self-report measure and to evaluate its psychometric properties in term of dimensionality, longitudinal invariance, and reliability in term of both internal consistency and test-retest reliability.

## Methods

### Participants and procedure

An Italian sample of 284 patients with localized prostate cancer who had undergone RARP were recruited at the European Institute of Oncology in Milan between July 2015 and July 2016. Patients were included in the study if they: 1) were diagnosed with localized prostate cancer, 2) were native Italian speakers, 3) referred to the Value Based Project and 4) had neither neurological nor psychopathological problems. They completed the questionnaire 45 days (T1) and 6 months (T2) after RARP surgery. Informed consent was provided and signed by each participant. The participation in the study was voluntary and at each moment, patients could withdraw their consent. The study was developed in accordance with the principles stated in the Declaration of Helsinki (59th WMA General Assembly, Seoul, 2008) and was approved by the Ethical Committee of the European Institute of Oncology.

### Language equivalence

Transcultural adaptation of the EPIC-26 survey in Italian was done using forward and backward translation by two experts [18]. One English native speaker translated the original English EPIC-26 version into Italian. Then, two expert psychologists assessed the consistency of the translation and approved the first version of the Italian EPIC-26. This version was pretested in a cognitive debriefing study with ten prostate cancer patients in order to assess its readability, understand ability, and comprehensibility. The cognitive debriefing was conducted by a psychologist. The time taken by each patient to complete the EPIC-26 was recorded. Patients then completed a cognitive debriefing task in which they asked about the clarity of the instructions and items, and the level of ease of response to each item.

Instructions (M = 4.80, ds = 0.632) and items (M = 4.96, ds = 0.08) were rated as clear on a five-point Likert scale (ranging from 1 = not at all to 5 = completely). Items were also rated as easy to complete (M = 4.96, ds = 0.07) on a five-point Likert scale (from 1 = not at all to 5 = completely). Only two patients asked for further information about the “13.b” item (“breast tenderness/enlargement”): they did not understand the meaning of the question and asked for more information. They did not know this side effect and were not able to visualise it as a possible consequence of the disease. Then, a second mother tongue speaker translated this version back into English language. The results of this back translation were virtually identical to the original English version.

### Statistical analysis

The psychometric properties were assessed using structural equation modelling in a sample of patients who had undergone Robot-Assisted Radical Prostatectomy (RARP). Specifically, we aimed at assessing the goodness of fit of the CFFM for the EPIC-26 using confirmatory factor analysis (CFA) and testing reliability. Then, longitudinal invariance was assessed to evaluate the ability of the EPIC-26 to reliably and validly measure its relevant constructs over time. Longitudinal invariance is a necessary requisite to assess stability and change of constructs over time since without invariance it is not possible to distinguish between true changes in outcomes over time and differences in the psychometric properties of the instrument.CFA with robust maximum likelihood (MLR) [19] was performed with Mplus 8.2 to evaluate the CFFM of the EPIC-26 separately at T1 and T2. Overall goodness-of-fit of the proposed models was evaluated assessing multiple indices of fit: the chi square test (*Χ*^2^), the root mean square error of approximation (RMSEA), the comparative fit index (CFI), the Tucker-Lewis index (TLI), and the standardized root mean square residual (SRMR). The model fit was firstly evaluated using the *Χ*^2^ statistic. However, because of its sensitivity to the sample size, other indices were also used [20]. Specifically, values above .90 for the CFI and TLI, a RMSEA below .06, and a SRMR below .08 indicate a good fitting model. The assessment of longitudinal invariance is a sequential process with seven specific steps. As reported in Table 1, configural, metric, scalar, and residual variances invariances were sequentially performed and followed by invariance of the latent factor variances, covariances, and means. The main assumption of configural invariance is that the same factor structure will hold in the two waves. Metric and scalar invariance respectively assume that factor loadings and item intercepts are equivalent across time. The assumption of residual invariance is that the residual variances of items are equivalent across time. Equality of residual variances implies equal reliability over time. Finally, the invariance of factor variances, covariances, and means assume respectively that latent factor variances, invariances, and means are equal across the two waves. The difference in CFI (i.e., ΔCFI) between a model and the subsequent level of invariance was considered to evaluate whether the hypothesis of invariance should be retained. Measurement invariance is indicated by a ΔCFI less than or equal to −.010 [21]. When invariance was not found, we tested partial invariance by checking modification index (MI). At each step, we identified all the non-invariant parameters by reviewing MI and then freely estimated these parameters across time. Analyses were performed using the full-information maximum likelihood estimator, which allows for inclusion of cases with partially missing data.

**Table 1:** The sequential process to assess longitudinal invariance

Model	Title	Description
A	Configural model	The factor structure is the same across waves
B	Metric model	A + all factor loadings are constrained to be equivalent across waves
C	Scalar model	B + all item intercepts are constrained to be equivalent across waves
D	Residual variances model	C + all residual variances of items are constrained to be equivalent across waves
E	Factor variances model	D + all latent factor variances are constrained to be equivalent across waves
F	Factor covariances model	E + all covariances among latent factors are constrained to be equivalent across waves
G	Factor means model	F + all latent factor means are constrained to be equivalent across waves

Internal consistency was assessed by computing respectively Cronbach’s alpha of each dimension in the two waves. Test-retest reliability was computed by considering intraclass correlations (ICCs). Specifically, ICCs (and their 95% confidence interval) were used to examine correlations between repeated measurements of each QoL dimensions obtained from the same patient at different times (i.e., T1 and T2). We used ICC Model 3 (i.e., two-way mixed effects, absolute agreement, single measure/rater) to quantify test-retest reliability [22, 23]. ICC values below 0.50 were considered to indicate poor reliability, from 0.50 to 0.75 moderate, from 0.75 to 0.90 good, and above 0.90 excellent reliability [24].

## Results

As shown in Table 2, participants had a median age of 63.4 ± 7.12 and a BMI of 26.6 ± 3.54. Two hundred and thirty-three men underwent radical prostatectomy with nerve-sparing (NS) surgical procedure (*N* = 159 with bilateral NS; *N* = 75 with unilateral NS), while the other 17.6% (50/284) of the sample undergone surgery without NS. The distribution of item responses was reported in Table 3.

**Table 2:** Sample characteristics

Age	63.4 ± 7.12
BMI	26.6 ± 3.54
Type of surgery	
Bilateral NS	159
Unilateral NS	75
Without NS	50

**Table 3:** The distribution of item responses

	T1		T2	
ITEMS	M	SD	M	SD
1	2,13	1,59	3,72	1,62
2	2,75	0,76	3,30	0,68
3	1,41	1,08	0,46	0,73
4a	1,92	1,26	0,90	1,07
4b	0,62	0,94	0,10	0,38
4c	0,20	0,58	0,01	0,13
4d	0,79	1,09	0,40	0,77
4e	1,90	1,21	1,08	1,14
6a	0,42	0,79	0,28	0,63
6b	0,34	0,72	0,21	0,59
6c	0,03	0,19	0,03	0,26
6d	0,02	0,12	0,03	0,19
6e	0,75	1,00	0,28	0,62
7	1,55	0,90	1,31	0,69
8a	1,58	0,94	1,82	1,08
8b	1,87	1,19	2,28	1,27
9	1,91	1,13	2,19	1,20
10	1,91	1,33	2,15	1,36
11	1,57	0,97	1,73	1,07
12	2,81	1,39	3,02	1,46
13a	1,12	0,47	1,25	0,73
13b	1,08	0,38	1,10	0,40
13c	1,56	0,91	1,56	0,99
13d	1,90	1,08	1,54	0,94
13e	1,38	0,78	1,34	0,80

At T1, the CFFM model displayed a good fit to data [SB *Χ*^2^ (265) = 553.092, *p* = .000; RMSEA = .055; CFI = .921; TLI = .911; SRMR = .067]. Similarly, the model showed an adequate fit also at T2 [SB *Χ*^2^ (265) = 605.020, p = .000; RMSEA = .060; CFI = .907; TLI = .894; SRMR = .061]. Specifically, all standardized factor loadings except the ones for items 13 and 23 are significant at T1. At T2, all standardized factor loadings are significant except the ones for items 7, 12, and 13.

Table 4 summarizes the sequential process of assessing measurement invariance by reporting fit indices of each model and the ΔCFI between them. In the first step, configural invariance was assessed. Specifically, fit indices attested that the CFFM had a good fit in both waves hold in the two waves [SB *Χ*^2^ (1105) = 1892.249, *p* = .000; RMSEA = .044; CFI = .913; TLI = .904; SRMR = .062]. Equivalence of the factor loading across waves was then examined in the metric invariance model. This model did not fit significantly worse than the configural model (ΔCFI = −.010) thus indicating that each item was related to the latent factor equivalently across waves. The scalar invariance model fitted significantly worse than the metric invariance one (ΔCFI = −.028). Subsequently, the MIs suggested that the intercept of items 14, 5, 25, 6 and 21 were the main sources of significant misfit and should be freely estimated across waves. After doing this, the partial scalar invariance model did not fit significantly worse than the metric invariance one (ΔCFI = −.009) and thus denoting that T1 and T2 had the same expected response for each item except for items 14, 5, 25, 6, and 21 at the same absolute level of the traits being measured. The residual variances invariance model fitted significantly worse than the partial scalar one (ΔCFI = −.012). The MIs suggested that the residual variances of items 13 and 22 should be freely estimated across the two waves. After doing so, the partial residual variances invariance model did not significantly fit worse than the previous invariance model (ΔCFI = −.005) and thus denoting that the amount of item variance not accounted by the latent factor was the same across the two waves except for items 13 and 22. After reaching partial measurement invariance, structural invariance was assessed by evaluating factor variances, factor covariances, and factor means invariance. The factor variance model did not fit significantly worse than the partial residual variances invariance model (ΔCFI = −.002) thus indicating equivalent variances or namely equal amounts of individual differences in QoL across the two waves. Results demonstrated the equivalence of relationships among the five latent factors across waves as indicated by a no significant decrease of model fit between the factor covariances invariance model and the previous model (ΔCFI = −.004). Finally, the factor means invariance model fitted significantly worse than the factor covariances model (ΔCFI = −.036). The MIs suggested that the means of the latent factors of urinary incontinence and urinary irritation should be freely estimated across the two waves. After doing so, the partial factor means invariance model did not significantly fit worse than the previous invariance model (ΔCFI = −.006) and thus denoting that only these two factors means were significantly different and decreasing over time.

**Table 4:** Results of the sequential process of assessing measurement invariance of the EPIC-26

Model	SB *Χ*^2^	df	p	RMSEA	CFI	TLI	SRMR	ΔCFI
Configural Invariance	**1892.249**	**1105**	**.000**	**.044**	**.913**	**.904**	**.062**	**–**
Metric Invariance	**2003.830**	**1125**	**.000**	**.047**	**.903**	**.895**	**.068**	**- .010**
Scalar Invariance	2279.990	1145	.000	.052	.875	.866	.071	- .028
Partial Scalar Invariance - Item 14	2225.603	1144	.000	.051	.881	.872	.071	- .022
Partial Scalar Invariance – Item 5	2196.445	1143	.000	.051	.884	.876	.070	- .019
Partial Scalar Invariance – Item 25	2155.922	1142	.000	.050	.888	.880	.070	- .015
Partial Scalar Invariance – Item 6	2126.187	1141	.000	.049	.891	.883	.069	- .012
Partial Scalar Invariance – Item 21	**2101.490**	**1140**	**.000**	**.048**	**.894**	**.886**	**.069**	**- .009**
Residual Variance Invariance	2232.515	1160	.000	.051	.882	.875	.078	- .012
Partial Residual Variance Invariance – Item 13	2219.857	1159	.000	.050	.883	.876	.075	- .011
Partial Residual Variance Invariance – Item 22	**2167.399**	**1158**	**.000**	**.049**	**.889**	**.882**	**.072**	**- .005**
Factor Variance Invariance	**2191.584**	**1163**	**.000**	**.050**	**.887**	**.881**	**.077**	**- .002**
Factor Covariance Invariance	**2231.046**	**1173**	**.000**	**.050**	**.883**	**.878**	**.078**	**- .004**
Factor Mean Invariance	2562.749	1178	.000	.057	.847	.841	.098	- .036
Partial Factor Mean Invariance – Urinary Incontinence	2383.217	1177	.000	.053	.867	.862	.081	- .016
Partial Factor Mean Invariance – Urinary Irritation	**2291.617**	**1176**	**.000**	**.051**	**.877**	**.872**	**.078**	**- .006**

The best fitting model for each of the seven steps of measurement invariance assessment is indicated in bold

Note: *SB* Satorra-Bentler Chi Square, *df* degree of freedom, *RMSEA* Root mean square error of approximation, *CFI* Comparative fit index, *TLI* Tucker-Lewis index (TLI), *SRMR* Standardized root mean square residual, *ΔCFI* Difference in CFI between models

**Fig. 2:**
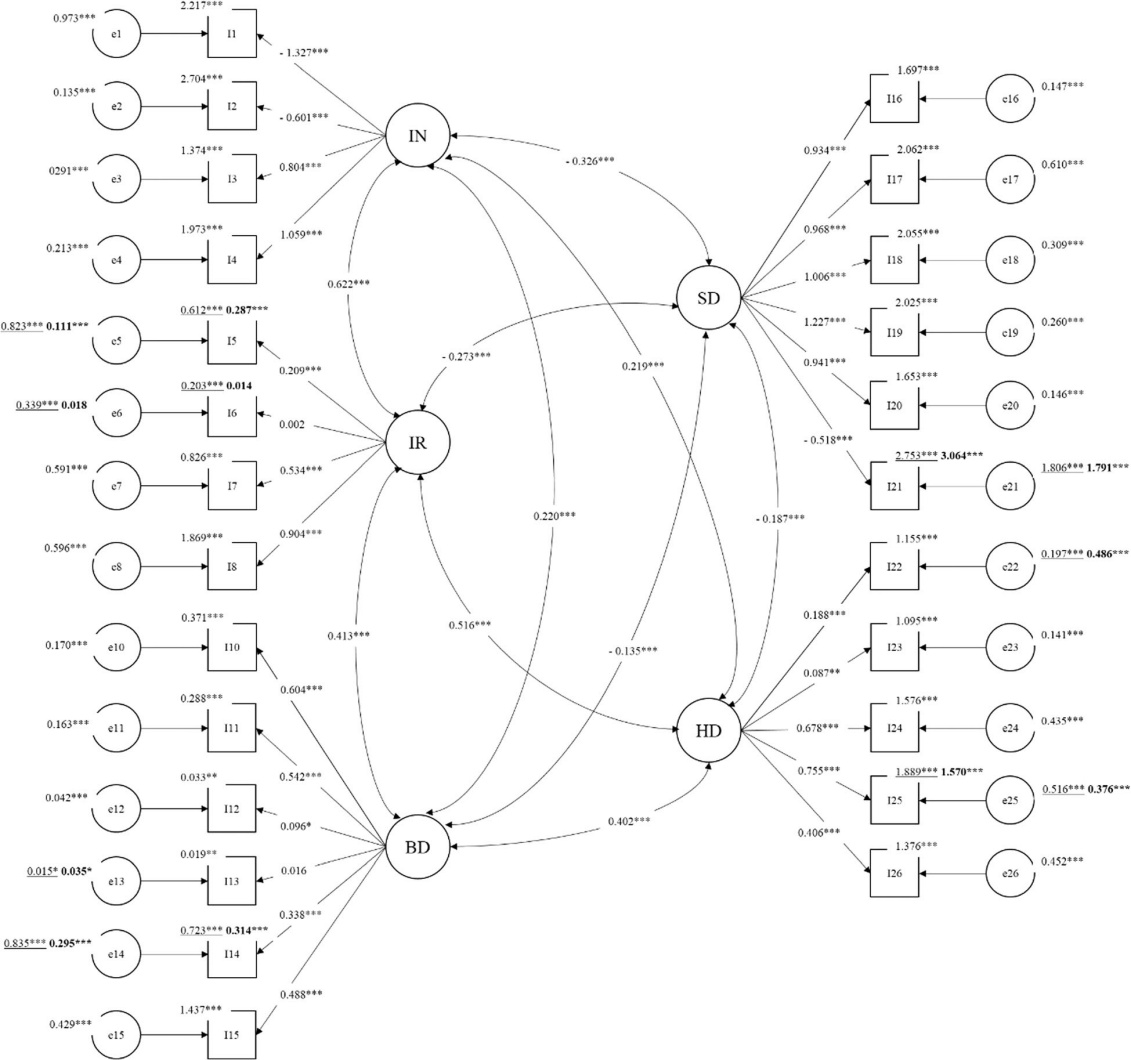
Standardized parameters of the final CFFM. Note: IN = Urinary incontinence; IR = Urinary irritation; BD = Bowel dysfunction; SD = Sexual dysfunction; HD = Hormonal dysfunction

This final model showed an adequate fit to data [SB *Χ*^2^ (1176) = 2291.617, *p* = .000; RMSEA = .051; CFI = .877; TLI = .872; SRMR = .078]. Standardized parameters of this model are reported in Fig. 2. All the standardized factor loadings are significant and above .30 in absolute value with the exception of items 5, 6, 12, 13, 22, and 23. Intercepts of items 5, 6, 14, and 25 decreased across waves while the intercept of item 21 increase from T1 to T2. Regarding residual variances, all non-equivalent items showed decrease of residual variance except for items 13 and 22 that showed an increase of residual variance over time. All the factor variances and covariances are equivalent across time attesting the structural stability of the EPIC-26 questionnaire. Factor correlation ranged between .187 and .622 in absolute value with the highest link between urinary incontinence and urinary irritation. Finally, three factor means (i.e., bowel, sexual, and hormonal dysfunctions) showed to be equivalent across waves; on the contrary, urinary incontinence and urinary irritation decrease from the first to the second wave.

Results of the reliability analysis attested the good internal consistency and test-retest reliability of each dimension (Table 5). Specifically, all Cronbach’s alphas could be classified as minimally acceptable (i.e., above .65) [25] except for low Cronbach’s alpha for hormonal dysfunction at T1 and urinary irritation at both waves. Urinary incontinence and sexual dysfunction display optimal reliability with values of Cronbach’s alpha above .80 in both waves. Finally, ICCs attested the good test-retest reliability of each dimension. Specifically, ICC for urinary incontinence could be classified as good, whereas the ICCs for the remaining dimensions could be considered as moderate.

**Table 5:** Cronbach’s alphas and ICC assessing internal consistency and test-retest reliability of the EPIC-26

Dimension	T1 Cronbach’alpha	T2 Cronbach’alpha	ICC correlation (95% CI)
Urinary incontinence	.886	.862	.764 (.717–.804)
Urinary irritation	.585	.518	.600 (.529–.662)
Bowel dysfunction	.699	.736	.536 (.458–.606)
Sexual dysfunction	.860	.902	.552 (−.088–830)
Hormonal dysfunction	.638	.700	.636 (.570–693)

## Discussion

This study represents the first attempt to assess the validity of the Italian version of the EPIC-26. The factor structure, longitudinal invariance and reliability of the Italian version of the EPIC-26 were investigated in a sample of Italian prostate cancer patients who had undergone RARP. Results of the CFA demonstrated that the proposed CFFM provided a good fit to data at both waves in these patients. These results support the usefulness and validity of computing separate scores for each of the five domains of urinary incontinence, urinary irritation, bowel dysfunction, sexual dysfunction, and hormonal dysfunction. The results of the reliability analyses attest the acceptable internal consistency and test-retest reliability of most of the EPIC-26 domains. The urinary irritation subscale is the only dimension showing a poor internal consistency with values of Cronbach’s alpha below the minimally acceptable cut-off at both waves. This result is consistent with previous empirical evidence regarding the weak reliability of this subscale in the Chinese version of the EPIC-26 [14]. Following suggestions by Lam and colleagues [14], the low reliability of this subscale may be determined by the high proportion of patients reporting no problem on the first two items (pain on urination and bleeding with urination) of this domain and a higher proportion of patients reporting moderate problems or incomplete emptying and need to urinate frequently during the day. Another possible explanation of this low reliability is the limited number of items in this domain compared with other domains (urinary incontinence, sexual, bowel, and hormonal dysfunction). Notably, the first two items measuring urinary irritation, alongside with item 2 (urinary control), item 13 (bloody stools), item 22 (hot flashes), and item 23 (breast tenderness) display a low factor loading below .30. Thus, all these items could be considered weak indicators of their respective dimensions. Further research is needed to identify more reliable indicators of urinary irritation in patients with prostate cancer by developing new ad-hoc items. Regarding test-retest reliability, all dimensions displayed at least moderate reliability.

Testing longitudinal measurement invariance is a pre-requisite for understanding whether changes in patients’ urinary incontinence, urinary irritation, bowel dysfunction, sexual symptomatology, and hormonal dysfunction over time reflect true changes in quality of life or rather changes in the psychometric properties or structure of the EPIC-26 over time. This study also demonstrated the good longitudinal invariance of the EPIC-26. This self-report measure was administered to the same sample of patients with prostate cancer who had undergone RARP in order to assess all the sequential steps of measurement invariance over time. Results demonstrated a full weak invariance of the EPIC-26 across time. Specifically, its entire factor loading is invariant over time and, thus, indicating that all of them are related to their respective domains equivalently across waves. We also demonstrated a partial strong invariance and a partial strict invariance of the EPIC-26 over time attesting respectively that the majority of the expected responses are equivalent over time and that the amount of item variance not accounted by the latent factor was the same across the two waves. The non-invariant thresholds of items 5, 6, 14, 21, and 25 suggested that patients evaluate these specific symptoms differently over time. Specifically, responses to these items revealed that patients who had undergone RARP reported a significant decrease over time of pain or burning on urination, bleeding on urination, bloody stools, and lack of energy. Conversely, they showed a significant increase of amount of problem related to their sexual function or the lack of sexual function.

Finally, after the partial strict invariance, results also attested the structural invariance of the EPIC-26 across the two waves. We found equivalence of factor variances and covariances over time suggesting respectively that the same amounts of individual differences in patients’ quality of life were found between T1 and T2 and that a strong structural stability exists among the five EPIC-26 domains over time. Moreover, the five EPIC-26 domains showed from moderate to strong stability across time. Finally, the results of the factor mean invariance demonstrated that the levels of bowel, sexual, and hormonal dysfunctions tend to be equal over time. On the other hand, self-reported levels of urinary incontinence and irritation significantly decreased from 45 days to 6 months after the RARP.

The large number of studies using this instrument (the 50- and 26-item versions) [10] and the high number of language translations [12–15, 26–31] make an Italian validation necessary. The Italian validation of the Expanded Prostate Cancer Index Composite – Short Form confirms its validity and reliability in measuring Quality of Life in prostate cancer patients over time. Beyond its reliability, the Short Form is easier than the longer version of the questionnaire, reducing administration burden with only 10 min for the compilation [10].

### Limitations

One of the main limitations of this study is the lack of other self-report measures of quality of life or patients’ well-being, which could be useful to better assess convergent and/or divergent validity of the EPIC-26. However, we did not include any other measures to minimize burden on such patients.

Moreover, the EPIC-26 was administered to patients who had undergone RARP only; the lack of other treatment types may affect the internal consistency. More precisely, our results may show poor internal consistency in the urinary irritation subscale because patients who had undergone RARP did not suffer from this side effect.

## Conclusion

To sum up, the current study attests the validity and reliability of the CFFM of the Italian version of the EPIC-26 in patients with prostate cancer undergone RARP. Thus, we suggest using five distinct domain scores of urinary incontinence, urinary irritation, bowel dysfunction, sexual symptomatology, and hormonal dysfunction on both clinical and research practice. The EPIC-26 is demonstrated to be a valid and reliable self-report measure of health-related quality of life for patients with prostate cancer.

## Data Availability

The data that support the findings of this study are available from the European Institute of Oncology, but restrictions apply to the availability of these data, which were used under license for the current study, and so are not publicly available.

## Abbreviations

CFA: Confirmatory factor analysis
CFFM: Correlated five-factor model
CFI: Comparative fit index
EPIC-26: Expanded Prostate Cancer Index Composite – Short Form
MLR: Robust maximum likelihood
PROs: Patient-reported outcomes
QoL: Quality of Life
RARP: Robot-Assisted Radical Prostatectomy
RMSEA: Root mean square error of approximation
SRMR: Standardized root mean square residual
TLI: Tucker-Lewis index
UCLA-PCI: University of California—Los Angeles Prostate Cancer Index
